# Verification of Data from Supersensitive Detector of Hydrosphere Pressure Variations

**DOI:** 10.3390/s23156915

**Published:** 2023-08-03

**Authors:** Grigory Dolgikh, Stanislav Dolgikh, Mikhail Ivanov

**Affiliations:** V.I. Il’ichev Pacific Oceanological Institute, Far Eastern Branch Russian Academy of Sciences, 690041 Vladivostok, Russia; dolgikh@poi.dvo.ru (G.D.); ivanov.mp@poi.dvo.ru (M.I.)

**Keywords:** supersensitive detector of hydrosphere pressure variations, sound velocity profiler with pressure and temperature sensors, sea waves

## Abstract

The paper describes experimental research and the results of these studies carried out in various bays of the Primorsky Territory of Russia using a supersensitive detector of hydrosphere pressure variations and a sound velocity profiler with pressure and temperature sensors. In all experiments, instruments, rigidly fixed to each other, were placed on the bottom at a depth of up to 10 m. Comparison of in-situ data from these instruments allowed us to experimentally calculate the coefficient of data conversion of the supersensitive detector of hydrosphere pressure variations when registering sea waves with periods ranging from several seconds to tens of minutes.

## 1. Introduction

Since 2000, employees of the Laboratory “Physics of the Geospheres” of the V.I.Il’ichev Pacific Oceanological Institute FEB RAS have been developing hydrophysical laser-interference receiving systems. Over the past two decades, several modifications of receiving systems based on the equal-arm Michelson interferometer with the use of semiconductor- and frequency-stabilized helium-neon lasers as light sources have been created. The most successful of them is the supersensitive detector of hydrosphere pressure variations [1]. This instrument has unique amplitude-frequency characteristics and allows measurements of hydrosphere pressure variations with nanolevel accuracy in the infrasonic and sonic bands in a wide dynamic range. The main characteristics of the supersensitive detector of hydrosphere pressure variations are: operating frequency range—from 0 (conditionally) to 1000 Hz; measurement accuracy—0.24 MPa; operating depths—up to 50 m.

This system was used in fundamental and applied research intended to obtain unique results in the wide frequency and dynamic ranges. In-situ data obtained from this instrument made it possible to study the eigen oscillations of the bays within Posyet Bay of the Sea of Japan [2], the interaction of wind waves with lower-frequency sea waves [3], the mechanisms of nonlinear hydrophysical disturbances’ occurrence in the range of gravity and infragravity sea waves [4], and many other natural phenomena.

Evaluation of the amplitude of sea waves recorded by the supersensitive detector of hydrosphere pressure variations was carried out on the basis of theoretical calculations. To verify theoretical calculations and expand the limits of the instrument’s application when registering hydrosphere pressure variations, we need to calculate the coefficient of conversion of the in-situ data of the supersensitive detector of hydrosphere pressure variations. For this purpose, a number of experiments have been carried out in the bays of the Primorsky Territory of Russia at various depths, where a Valeport Mini SVP sound velocity profiler with pressure and temperature sensors was submerged to the bottom with this system. In order to make the conversion factor for recalculation of amplitude values more accurate, a number of experiments were carried out in the bays of the Primorsky Territory of Russia at various depths. A Valeport Mini SVP sound velocity profiler with pressure and temperature sensors was submerged to the bottom together with the above instrument. Comparison of the data from the created device and from similar certified devices allows one to evaluate the quality of the obtained data. Many developers of offshore systems [5,6,7] use these methods. In other experiments, we used commercially produced devices in the same housing as those of our creation [8,9]. Recently, a similar technique has been used in the application of neural networks [10,11,12]. All modern instruments significantly improve the results of the experimental studies and, in combination with modern numerical models and neural networks, make it possible to find new patterns in marine wave processes [13,14]. The obtained in situ data allow us to take a fresh look at the discovery of their sources, both natural and anthropogenic [15,16].

To study sea waves, it is necessary to use equipment capable of recording variations in water level fluctuations with high accuracy in a wide frequency range. It is these systems that allow conducting studies by direct methods and obtaining the most accurate results. These methods will make it possible to evaluate qualitatively not only wind waves, tidal oscillations, and extreme waves but also the subtle nonlinear effects of their behavior. First of all, they include rogue waves, the amplitudes of which are more than 2.8 times higher than the amplitudes of regular sea waves [17,18], meteorological tsunamis [19,20], storm surges [21,22], and other catastrophic phenomena. In our work, to quantify the amplitudes of sea waves, we used verification of the data from the supersensitive detector of hydrosphere pressure variations through comparison with the data from the pressure sensor of the Mini SVP sound velocity profiler from Valeport [23]. These systems were set up as an integrated unit and were installed on the bottom at depths of up to 10 m in various bays of the Primorsky Territory for several days. Then, sea waves with the same periods were selected from the in situ data array, and their amplitudes were compared. As a result, the data conversion coefficient of the supersensitive detector of hydrosphere pressure variations was calculated, depending on the installation depth.

The paper consists of three sections and conclusions: Receiving Systems and Description of the Experiment; processing and analysis of experimental data; calculation of data conversion coefficient. The Receiving Systems and Description of the Experiment section provides a brief description of the measuring instruments and the places where they were installed. The following section describes the principle of selection and processing of the in situ data of the supersensitive detector of hydrosphere pressure variations and the pressure sensor of the Valeport Mini SVP sound velocity profiler. Next, the analysis of these data and the conversion coefficient of the laser interference instrument are calculated. In Conclusions, the results and conclusions of the article are presented.

## 2. Receiving Systems—Description of the Experiment

**Figure 1:**
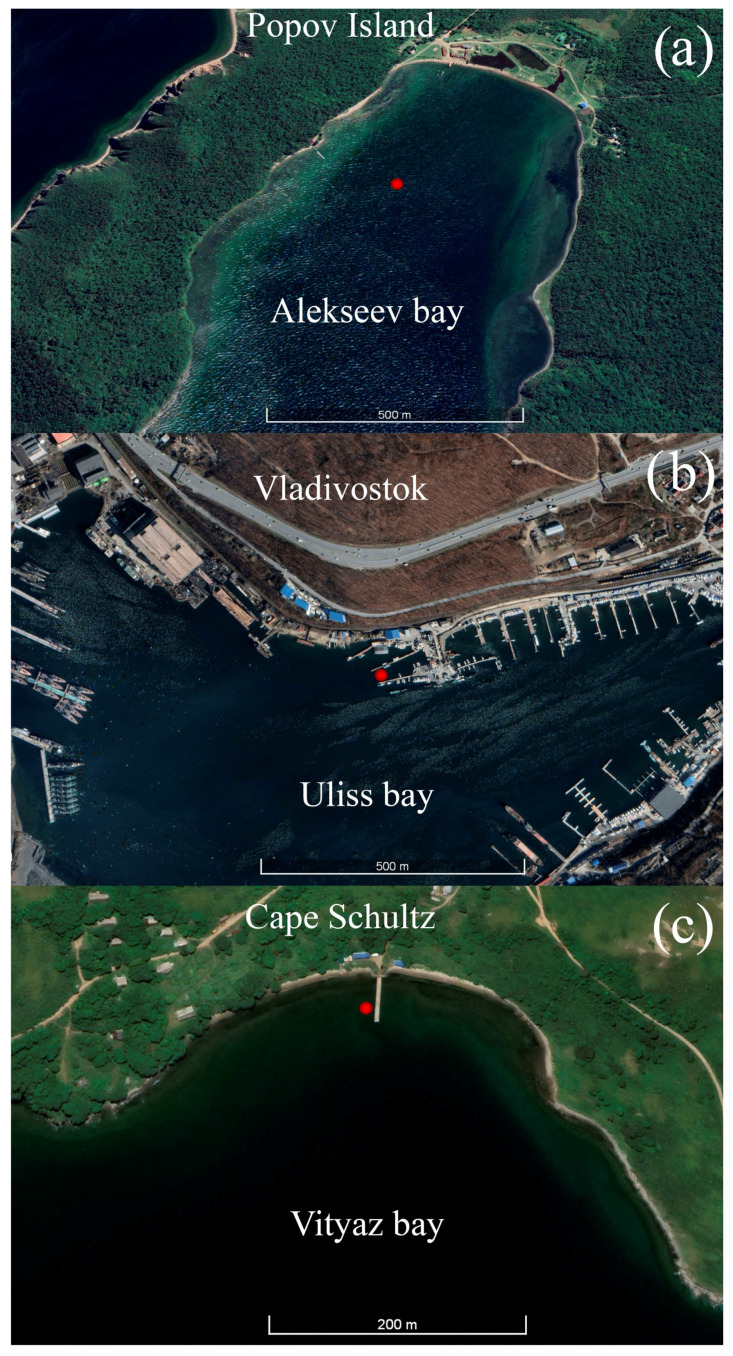
The instrument installation locations (red dot) in Alekseev bay (**a**), Uliss bay (**b**) and Vityaz bay (**c**).

Three experiments were carried out in different bays to calculate the data conversion coefficient of the supersensitive detector of hydrosphere pressure variations. The first experiment was conducted in Alekseev Bay of Popov Island, where the instruments were installed at a depth of 8 m on 24 June 2021 (Figure 1a). Then the instruments were installed in Ulysses Bay (Vladivostok) at a depth of 7 m in the period from 6 to 13 July 2021 (Figure 1b). The third experiment was conducted in Vityaz Bay, with the installation of the instruments at a depth of 5 m in the period from 30 June to 1 July 2022 (Figure 1c). In all three figures, the installation locations of the instruments are shown with a red circle.

**Figure 2:**
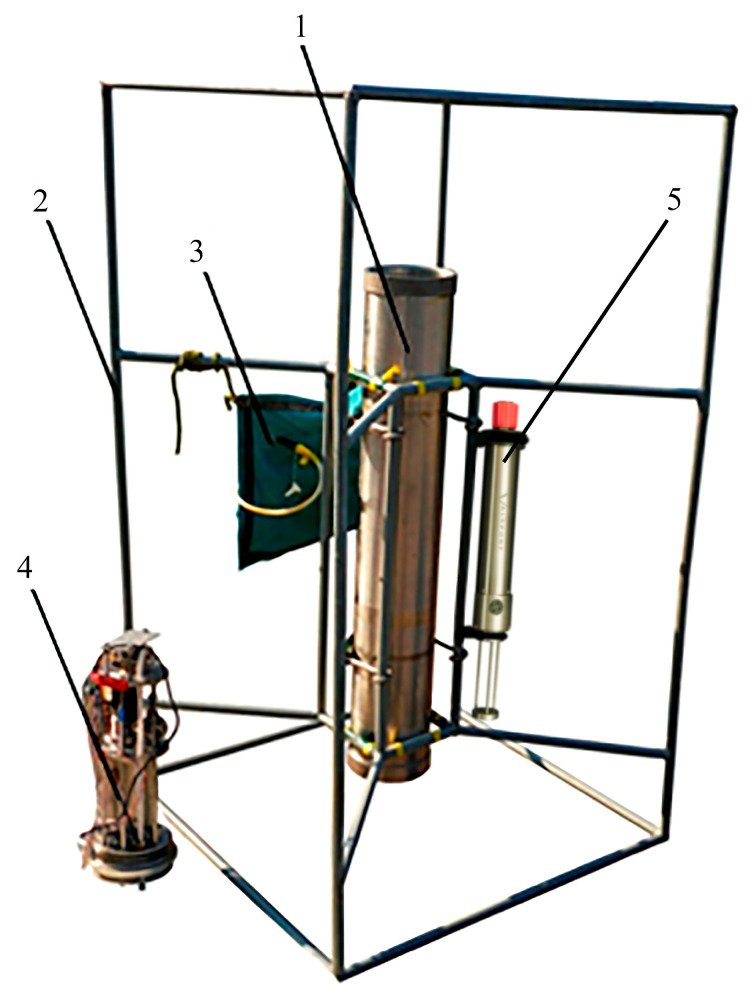
Supersensitive detector of hydrosphere pressure variations and Valeport Mini SVP sound velocity profiler.

In the course of experimental work, the sound velocity profiler was fixed inside the protective cage of the supersensitive detector of hydrosphere pressure variations. Figure 2 shows a photograph of the instrument’s location. In the figure, the following numbers are shown: 1—body of the laser-interference device; 2—protective cage; 3—air-filled container necessary to set the measuring membrane to the neutral position; 4—optical part of the supersensitive detector of hydrosphere pressure variations; 5—Valeport Mini SVP sound velocity profiler with pressure and temperature sensors, fixed in the protective cage.

**Figure 3:**
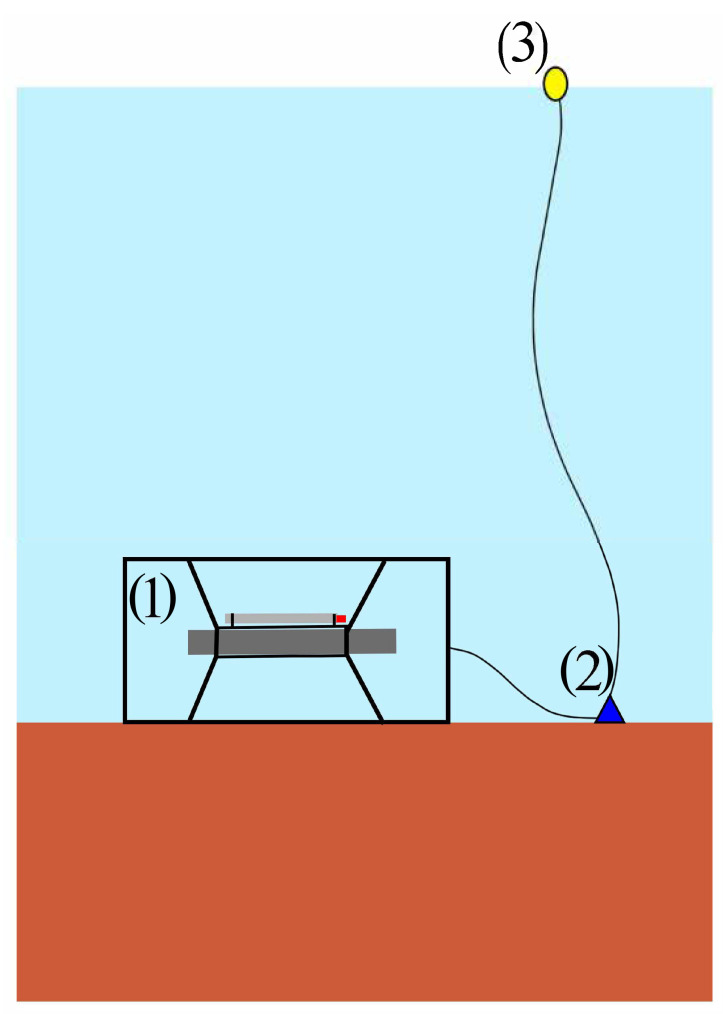
The installation schemes.

The supersensitive detector of hydrosphere pressure variations, with the Valeport Mini SVP sound velocity profiler attached to it, was installed on the bottom. In all three experiments, the installation scheme was the same, as shown in Figure 3. The instruments in a protective cage (1) were installed horizontally on the bottom. The protective cage prevents the instruments from sinking into the mud or sand on the bottom. Then a weight (2) was fixed to the protective cage, and a signal buoy (3) was lowered to the bottom. The use of weight is necessary to exclude the influence of surface sea waves on instrument readings. The oscillations transmitted from the signal buoy along the rope are damped by the weight. This arrangement of instruments makes it easy to lower and lift the instruments and to determine the installation location by the signal buoy.

**Figure 4:**
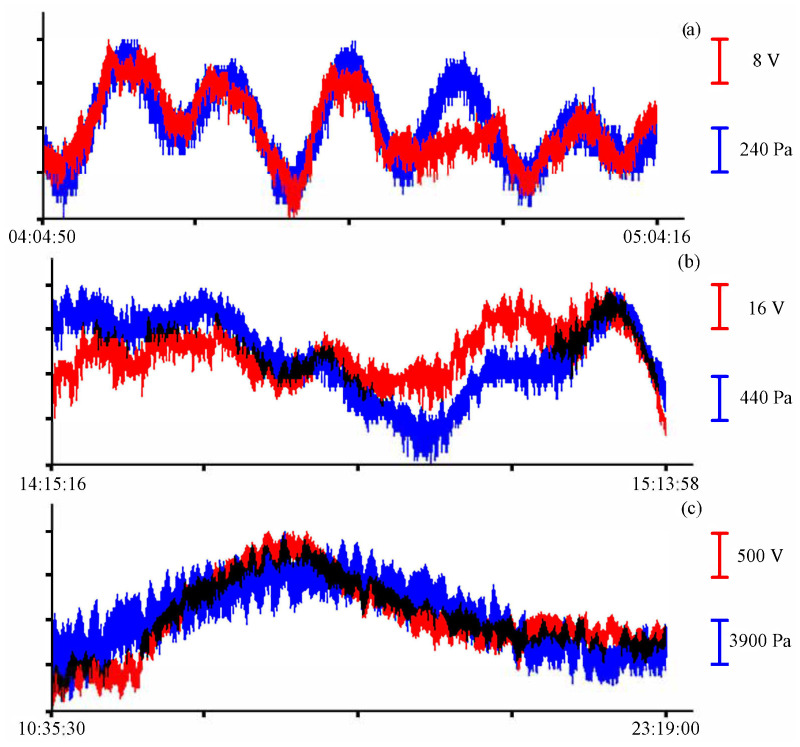
Synchronous records of the supersensitive detector of hydrosphere pressure variations (red) and the Valeport Mini SVP sound velocity profiler (blue) in Alekseev bay (**a**), Uliss bay (**b**) and Vityaz bay (**c**).

The sensitive element of the laser interference device is a round membrane rigidly fixed around the perimeter. Variations in hydrosphere pressure act on the membrane from one side, changing in proportion to sea waves. On the inside, the membrane is a part of the equal-arm Michelson interferometer located in the instrument. At the moment of reaching the working depth, the membrane is set to the neutral position by pumping air from the tank into the compensation chamber. Under the influence of variations in hydrosphere pressure, the membrane sags to one side or the other from its neutral position. The digital recording system installed inside the instrument is adjusted in such a way as to maintain the maximum brightness of the interference pattern unchanged. Deflection of the membrane also leads to a change in the length of the measuring arm of the laser interferometer. This, in turn, leads to a change in the brightness of the interference pattern. To maintain maximum brightness, voltage is applied to the piezoceramic compensation cylinder, which is a part of the interferometer, and it leads to a change in the length of the reference arm. The alignment of the interferometer arms allows for keeping the interference pattern at maximum brightness. The change in voltage applied to the piezoceramic cylinder is recorded by a digital registration system and transmitted to a computer. More details on the operating principle, design features, and assessment of the accuracy of measuring variations in hydrosphere pressure with laser-interference devices are described in [1]. After preprocessing, the experimental data from the supersensitive detector of hydrosphere pressure variations are formed into one hour-long file with a sampling rate of 800 Hz. This sampling frequency was chosen in accordance with the characteristics of the pressure sensor installed in the sound velocity profiler, which allows recording hydrosphere pressure variations with a maximum frequency of 8 Hz. However, with such a recording frequency, the sound velocity profiler can record continuously for just over 24 h. Therefore, to compare these instruments, we selected record fragments of shorter duration. Before installation, all instruments were synchronized. Figure 4 shows, as an example, synchronous fragments of the instruments records.

As a result of comparing the data, we obtained one-hour records of the supersensitive detector of hydrosphere pressure variations and the pressure sensor of the Valeport Mini SVP sound velocity profiler for Alekseev and Ulysses bays for 24 June and 9 July 2021, and a record of more than 12 h when instruments were installed in Vityaz Bay for 30 June 2022.

## 3. Processing and Analysis of Experimental Data

To calculate the conversion factor, let us compare the synchronous records of the instruments separately for each of the bays. Since the recording of pressure variations by the sensor of the Valeport Mini SVP sound velocity profiler was carried out at a frequency of 8 Hz and the recording of hydrosphere pressure variations by the laser interference devices was carried out at a frequency of 800 Hz, we need to bring them to the same frequency. To do this, we will filter the initial data of the supersensitive detector of hydrosphere pressure variations with a low-pass filter with a 3000-point Hamming window and decimate with averaging to the frequency of 8 Hz. Next, we will analyze the records obtained the instruments.

**Figure 5:**
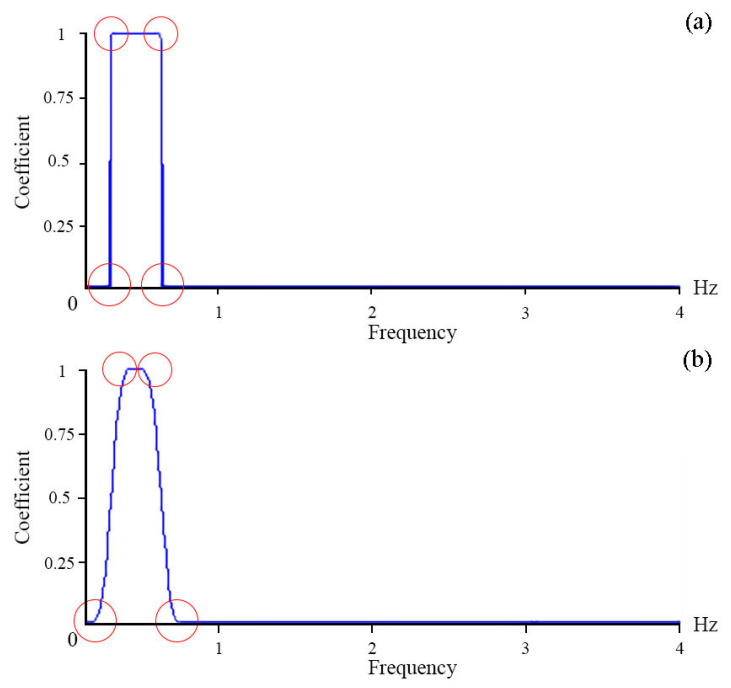
The amplitude-frequency response of the filter with window lengths of 4000 (**a**) and 100 (**b**). Red circles—boundary areas.

To single out lower-frequency oscillations, a band-pass filter with a Hamming window [24] will be applied. To select oscillations with periods from 8 s (0.125 Hz) to 5 min (0.003 Hz), the window length will be 4000, and to select oscillations with long periods, from 8 min (2 MHz) to 20 min (0.8 MHz), the window length will be 15,000. The choice of such window lengths is related to the frequency of the original signal and the shape of the filter. Figure 5 shows the forms of the amplitude-frequency response of the filter with window lengths of 4000 (a) and 100 (b). We can see from the figure that, with a greater length, its boundaries are sharper. This allows us to select a narrower filter band and eliminate unwanted frequencies. The use of a 15,000-length filter for lower-frequency oscillations is determined by the frequency of the original signal. At the frequency of 8 Hz (8 measurements per 1 s), for oscillations with periods of 20 min, there will be 9600 measurements. For such periods, the window length of 4000 will not be sufficient for accurate filtering.

### 3.1. Alekseev Bay

**Figure 6:**
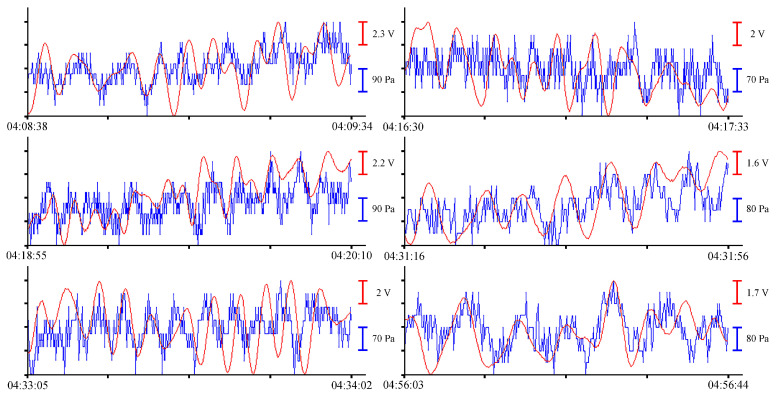
Synchronous records of wind waves from the supersensitive detector of hydrosphere pressure variations (red) and the Valeport Mini SVP sound velocity profiler (blue) of 24 June 2021.

Let us analyze the records of two instruments submerged to a depth of 8 m on 24 June 2021. From the entire spectrum of records of sea waves, we will single out oscillations with periods from several seconds (wind waves) to several minutes (infragravity sea waves, eigen oscillations of bays). To single out wind waves with periods ranging from 2.5 to 7 s, we will not filter preprocessed record files. Figure 6 shows examples of synchronous records of wind waves from the laser interference meter (red line) and the pressure sensor of the sound velocity profiler (blue line).

**Figure 7:**
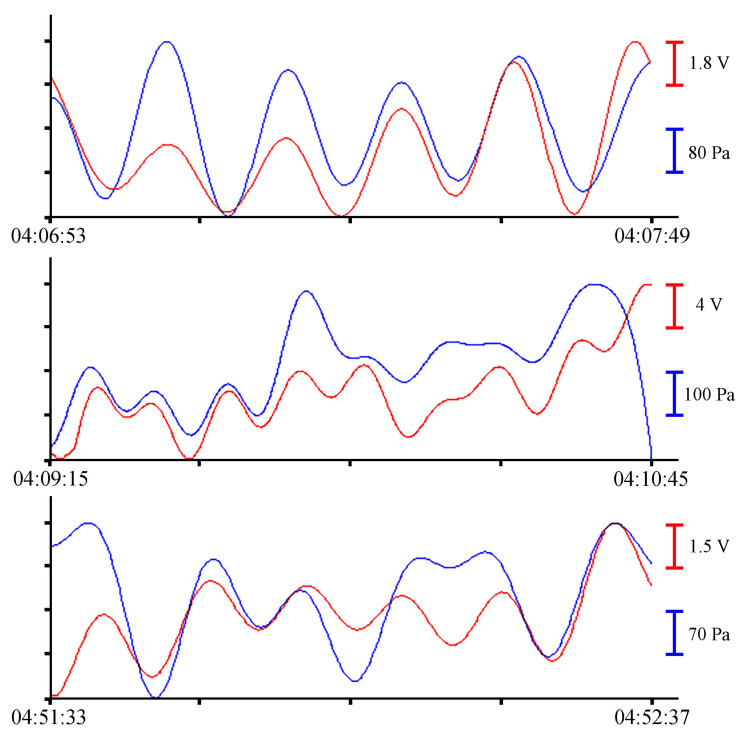
Filtered synchronized records of the supersensitive detector of hydrosphere pressure variations (red) and the Valeport Mini SVP sound velocity profiler (blue) of 24 June 2021.

In the records with a duration of about 1 min, wind waves with different periods are singled out. At different time intervals with different wind intensities, the amplitudes of these oscillations are also different. The analysis of the magnitudes of these amplitudes will be presented below. To select oscillations with periods from 10 to 16 s, let us filter the record files. We filter the pre-processed data files with a bandpass filter with a 4000-point Hamming window in the range of periods from 8 s to 1 min. After filtering, we select several fragments of records for analysis. Figure 7 shows filtered fragments of records as an example.

**Figure 8:**
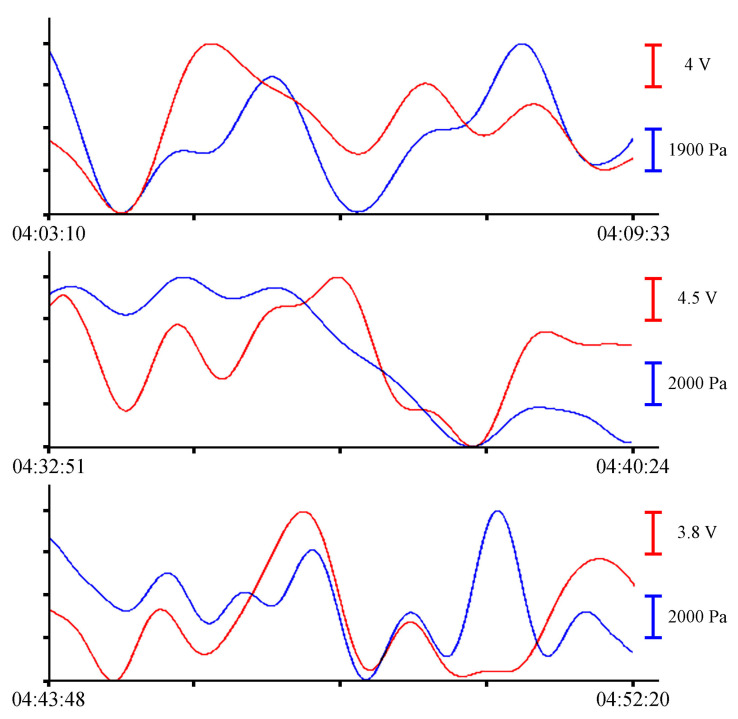
Filtered synchronized records of the supersensitive detector of hydrosphere pressure variations (red) and the Valeport Mini SVP sound velocity profiler (blue) of 24 June 2021.

All records with a duration of about 1 min, shown in Figure 7, contain oscillations with periods from 10 to 16 s. Further analysis of sea waves will be carried out in the range of periods of infragravity waves. To do this, we will filter the records with a bandpass filter with a 4000-point Hamming window in the range of periods from 1 to 8 min without decimation. As a result, oscillations with periods ranging from 1 to 5 min can be singled out from the entire spectrum of sea waves recorded by measuring instruments. The resulting fragments of the records are shown in Figure 8.

**Figure 9:**
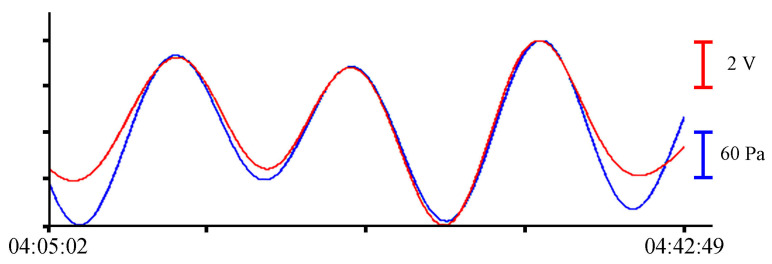
Filtered synchronized records of eigen oscillations of the bay from the supersensitive detector of hydrosphere pressure variations (red) and the pressure sensor of the Valeport Mini SVP sound velocity profiler (blue) of 24 June 2021.

All filtered synchronous records of the instruments with durations of 6 to 9 min, shown in Figure 8, contain oscillations with minute periods. Since we took a record of about an hour for analysis, there are not many such fragments. There are even fewer oscillations with periods corresponding to the eigen oscillations of the bay where the instruments were installed. To visualize these oscillations, we will filter the initial files with a bandpass filter with a 15,000-point Hamming window in the range of periods from 8 to 20 min. The resulting records are shown in Figure 9.

Oscillations with periods of about 10.5 min, corresponding to eigen oscillations of Alekseev Bay, were singled out in the filtered record of the instruments [25]. In Figure 6, Figure 7, Figure 8 and Figure 9, the record from the pressure sensor of the Valeport Mini SVP Sound velocity profiler is shown in blue, and the record from the supersensitive detector of hydrosphere pressure variations is shown in red. The x-axis scales are shown on the right of each curve with the corresponding color.

### 3.2. Ulysses Bay

**Figure 10:**
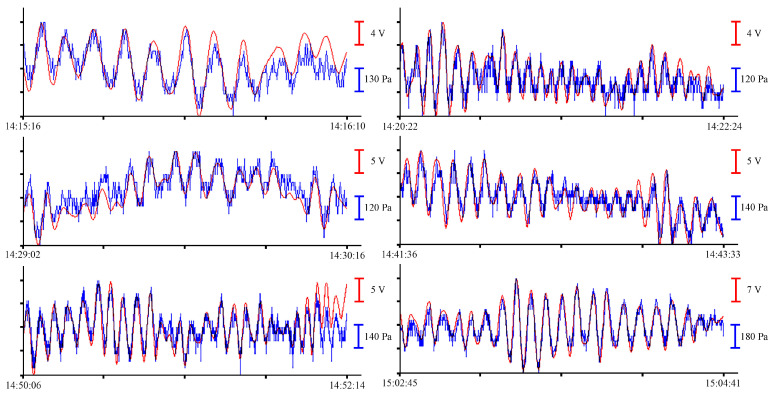
Synchronous records of wind waves from the supersensitive detector of hydrosphere pressure variations (red) and the Valeport Mini SVP sound velocity profiler (blue) of 9 July 2021.

The next experiment was carried out in Ulysses Bay, Vladivostok. The instruments were installed at a depth of 7 m from 6 July to 13 July 2021. Due to the limited recording capabilities of the sound velocity profiler and the supersensitive detector of hydrosphere pressure variations coming into operational mode, as in the previous case, for analysis we will select records with a duration of about an hour. Figure 10 shows synchronous records of the laser interference device and the sound velocity profiler.

**Figure 11:**
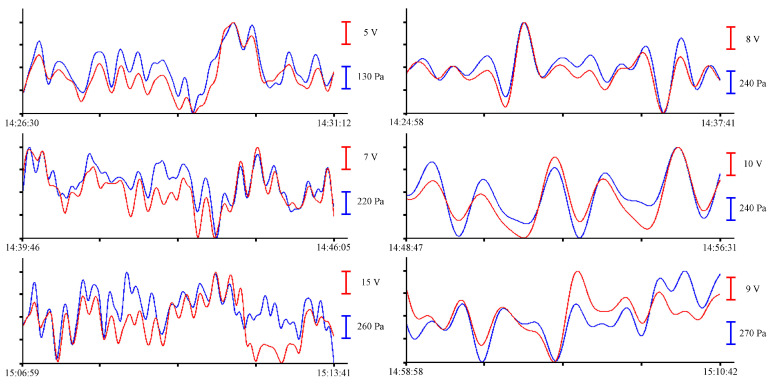
Filtered synchronized records of the supersensitive detector of hydrosphere pressure variations (red) and the Valeport Mini SVP sound velocity profiler (blue) of 9 July 2021.

On the synchronous fragments of the instruments’ records shown in Figure 10, with durations of 1 to 2 min, there are wind waves with periods of 2.5 to 6 s. The period and amplitude of wind waves change in accordance with the speed of the wind affecting the water surface. To single out oscillations with long periods, we filter the record files with a bandpass filter with a 4000-point Hamming window in the frequency range from 8 s to 1 min. After filtering, we select several fragments of records for analysis. Figure 11, on the left, shows the filtered fragments of records.

**Figure 12:**
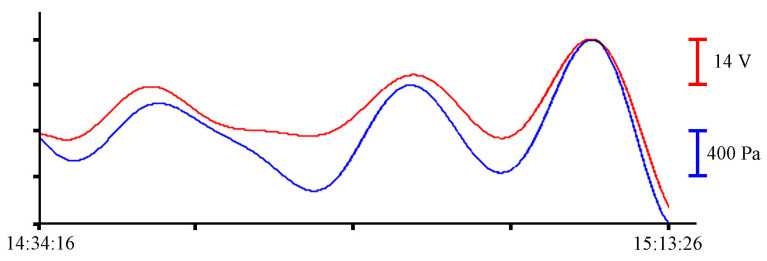
Filtered synchronized records of eigen oscillations of the bay from the supersensitive detector of hydrosphere pressure variations (red) and the pressure sensor of the Valeport Mini SVP sound velocity profiler (blue) of 9 July 2021.

All the records shown in Figure 11, left, with a duration of more than 5 min, contain oscillations with periods ranging from 10 to 25 s. Figure 11, right, shows records of the instruments, filtered by a bandpass filter with a 4000-point Hamming window in the range of periods ranging from 1 to 8 min without decimation. As a result, oscillations with periods from 1 to 6 min can be singled out from the entire spectrum of sea waves recorded by measuring instruments. To single out the eigen oscillations of Ulysses Bay, Vladivostok, we apply a bandpass filter with a 15,000-point Hamming window in the range of periods from 8 to 20 min to the initial record. The resulting records are shown in Figure 12.

When analyzing the filtered instrument record, three harmonics were singled out, corresponding to eigen oscillations of the bay where the instrument is installed [25]. In Figure 10, Figure 11 and Figure 12, as in the previous figures, the record of the pressure sensor of the Valeport Mini SVP sound velocity profiler is shown in blue, and the record of the supersensitive detector of hydrosphere pressure variations is shown in red.

### 3.3. Vityaz Bay

**Figure 13:**
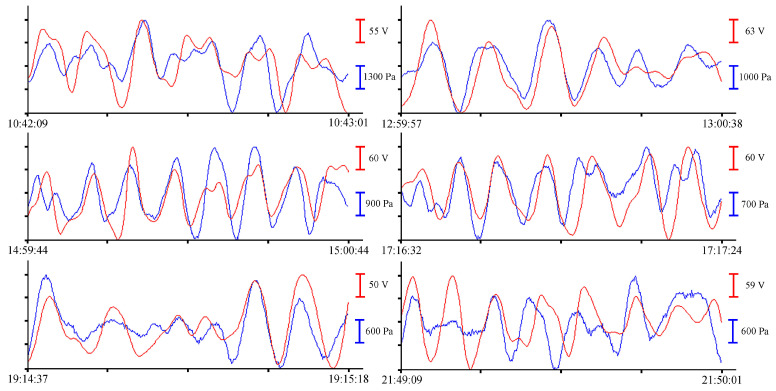
Synchronous records of wind waves from the supersensitive detector of hydrosphere pressure variations (red) and the Valeport Mini SVP sound velocity profiler (blue) of 30 June 2022.

In the summer of 2022, a similar experiment with the installation of these instruments was held in the south of the Primorsky Territory, in Vityaz Bay. This time, the instruments were installed at a depth of 5 m. As a result of preprocessing, a synchronous record was obtained for more than 12 h. Let us select several fragments of the records of the supersensitive detector of hydrosphere pressure variations and the pressure sensor of the Valeport Mini SVP sound velocity profiler to estimate the amplitudes of wind waves. In Figure 13, synchronous fragments of the instruments’ records are shown as an example.

**Figure 14:**
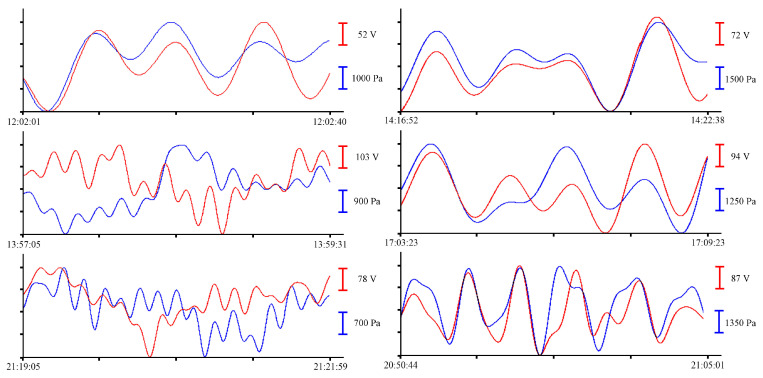
Filtered synchronized records of the supersensitive detector of hydrosphere pressure variations (red) and the Valeport Mini SVP sound velocity profiler (blue) of 30 June 2022.

All graphs shown in Figure 13 contain oscillations with periods ranging from 3 to 4 s, corresponding to the wind waves of the bay. The small range of changes in wind waves recorded during this experiment is due to the fact that the instruments were installed at the southern edge of the bay, and in summer, southerly winds prevail in this region. As in previous experiments, bandpass filters with different characteristics were applied to the initial record to single out oscillations with periods ranging from 10 s to several minutes. As a result of applying a bandpass filter with a 4000-point Hamming window ranging from 8 s to 1 min, oscillations with periods from 10 to 14 s are singled out. When applying a bandpass filter with a Hamming window of the same length in the range from 1 to 8 min, oscillations with periods from 1 to 6 min are distinguished. Figure 14 shows the filtered records of the supersensitive detector of hydrosphere pressure variations and the pressure sensor of the Valeport Mini SVP sound velocity profiler. On the left, after applying a bandpass filter from 8 s to 1 min, and on the right, after applying a bandpass filter from 1 to 8 min.

**Figure 15:**
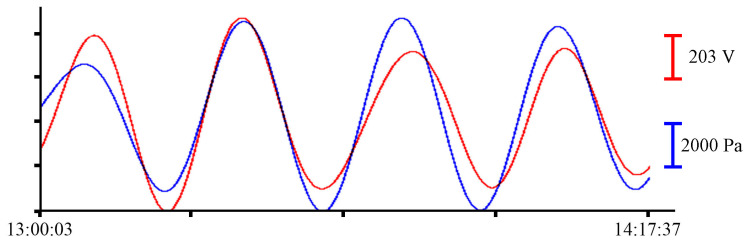
Filtered synchronized records of eigen oscillations of the bay from the supersensitive detector of hydrosphere pressure variations (red) and the pressure sensor of the Valeport Mini SVP sound velocity profiler (blue) of 30 June 2022.

To single out the eigen oscillations of the bay in which the instrument was installed, we filtered the record shown in Figure 4 with a bandpass filter with a 15,000-point Hamming window in the range of periods from 15 to 30 min. Application of such filter boundaries is related to the period of eigen oscillations in Vityaz Bay, which, according to the data given in [2], is slightly less than 20 min. Figure 15 shows an example of a filtered synchronous record of the instruments. As in all previous cases, the blue curve corresponds to the record from the pressure sensor of the Valeport Mini SVP sound velocity profiler, and the red curve corresponds to the recording of the supersensitive detector of hydrosphere pressure variations.

To calculate the coefficient of data conversion of the supersensitive detector of hydrosphere pressure variations from volts to pascals, we will use the amplitudes of sea waves with periods from wind waves to eigen oscillations of bays.

## 4. Calculation of Data Conversion Coefficient

To calculate the data conversion coefficient of the supersensitive detector of hydrosphere pressure variations for each of the periods of sea waves, we select several fragments with the same harmonics. Taking into account that the instruments in the bays were located at different depths, let us check whether there is a dependence of this coefficient on the depth of the instrument installation and on the period of sea waves. Let us start calculations with the smallest immersion depth in Vityaz Bay for 30 June 2022. The range of periods of wind waves during the experiment was from 3 to 4 s. Let us choose several wave packets with different periods but the same amplitudes. After selecting synchronous records of wave packets from both instruments, we estimate the value of the amplitudes. The obtained data are presented in Table 1, where T is the period of waves in the packet, U is the amplitude of the wave packet according to the data from the supersensitive detector of hydrosphere pressure variations, P is the amplitude of the wave packet according to the data from the pressure sensor of the Valeport Mini SVP sound velocity profiler, and k is the ratio of the amplitudes from two instruments.

Let us look at the amplitudes of swell waves recorded by measuring instruments in Vityaz Bay. These waves are not generated in the bay, but come from the Sea of Japan. The periods of the recorded waves in this experiment range from 10 to 14 s. As in the previous case, several trains of waves with different periods were selected and their amplitudes estimated. Table 2 lists the periods and amplitudes of ten selected wave trains.

Infragravity waves will be considered next. For calculations, we selected wave packets with periods ranging from 1 to 6 min. For each of the selected wave packets, we estimated the amplitudes, which, together with the wave periods, are presented in Table 3.

Next will be the eigen oscillations of Vityaz Bay and oscillations close to them. These are sea waves with periods from 15 to 19 min, which are typical for eigen oscillations of bays located not far from the instrument installation site and the Posyet Bay itself. Due to the large period, the amplitudes of these waves will be estimated from individual waves. The results are shown in Table 4.

The next experiment was held in Ulysses Bay in Vladivostok at a depth of 7 m. As in the previous case, we consider sea waves with periods ranging from several seconds to ten minutes. The first type of oscillation under consideration will be wind waves created by the impact of wind on the water surface of the bay itself. Table 5 shows the values of periods and amplitudes of wave packets with periods from 2 to 6 s. The data in the table are sorted by the length of the periods.

Let us consider the following group of waves recorded by the laser interference device and the pressure sensor of the sound velocity profiler. These are waves with periods ranging from 16 to 25 s. The values of the periods during the experiment were two times higher than the values of similar periods in the other two bays because, shortly before the work started, a cyclone passed over the water area. This cyclone generated waves with such periods in the Sea of Japan. Table 6 shows the periods and amplitudes of these wave packets recorded by the measuring instruments.

Let us consider the infragravity waves recorded in Ulysses Bay, with periods ranging from 1 to 6 min. For analysis, ten fragments of the records of the supersensitive detector of hydrosphere pressure variations and the pressure sensor of the Valeport Mini SVP sound velocity profiler were selected. The obtained values of periods and amplitudes are shown in Table 7.

The duration of synchronous recordings of the instruments during work in Ulysses Bay was about an hour. Only five waves with periods close to the periods of eigen oscillations in the bay could be singled out from this record. Table 8 shows the obtained values of periods and amplitudes.

The final experiment was held in Alekseev Bay, Popov Island. The depth of the instrument installation was 8 m. For this bay, we also chose sea waves with periods ranging from wind waves to eigen oscillations of the bays. Let us analyze the wind and sea waves generated in the bay. Their periods during the measurements ranged from 2.5 to 7 s. Let us choose ten trains of waves with such periods and estimate their amplitudes from the instrument readings. The results obtained are shown in Table 9.

As for the two previous bays, we will consider swell waves that enter the bay from outside. The periods of these waves at the time of registration ranged from 10 to 14 s. Table 10 shows the periods and amplitudes of these waves for ten packets.

Let us single out waves with periods from 1 to 12 min from the entire spectrum of sea waves. The values of the periods of infragravity waves and their amplitudes are presented in Table 11. The values of the amplitudes of waves with periods from 9 to 12 min are shown in Table 12.

**Figure 16:**
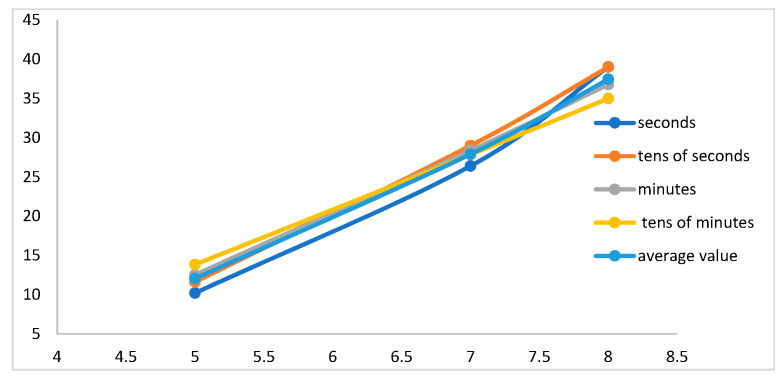
Graphs of the dependence of the data conversion coefficient of the supersensitive detector of hydrosphere pressure variations on the depth of immersion.

Let us calculate the data conversion coefficient of the supersensitive detector of hydrosphere pressure variations from the data given in the tables. To do this, we calculate the values of the average coefficient for each type of wave. Thus, for wind waves, when the instruments were installed at a depth of 5 m, the average value was 9.8 Pa/V, and when the instrument was placed at a depth of 7 m, the average value of the coefficient was 26.4 Pa/V. An increase in this value is also observed with an increase in the immersion depth; at the depth of 8 m, it was 38.8 Pa/V. When we considered the amplitudes of sea waves with periods from 10 to 25 s, the value of the conversion coefficient also increased with increasing immersion depth. For the depth of 5 m, it was 12 Pa/V, at the depth of 7 m, the value was 28.9 Pa/V and when the instruments were immersed to the depth of 8 m, this coefficient was 39.8 Pa/V. A similar situation is observed when analyzing the values of the ratio of the amplitudes of infragravity waves. The average value of the data conversion factor for depths of 5, 7, and 8 m is 12.5, 28.3, and 36.9 Pa/V, respectively. Waves with periods ranging from 9 to 20 min are no exception. When calculating the average values of the ratio of the amplitudes of these waves, an increase in the coefficient with increasing depth is also observed. When the instruments were immersed to a depth of 5 m, the coefficient was 12 Pa/V, and at depths of 7 and 8 m, the coefficient was 28.8 Pa/V and 36 Pa/V. Figure 16 shows the dependence of the average value of the data conversion coefficient of the supersensitive detector of hydrosphere pressure variations on the depth of immersion for each type of sea wave.

These calculations allow us to estimate the values of pressure variations recorded by the supersensitive detector of hydrosphere pressure variations. Calculations have shown that with an increase in the depth of immersion, the conversion coefficient also increases. Thus, with immersion to the depth of 5 m, the average value of the coefficient is 11.6 Pa/V; with immersion to the depth of 7 m, it is 28.1 Pa/V; and at the depth of 8 m, its value is 38 Pa/V. We can see from the obtained values and graphs that the coefficient does not change according to the linear law, and three immersion points are not enough to determine it at other depths. The dispersion of the obtained average values of the conversion coefficients for all types of sea waves at a depth of 5 m is 4.1, at a depth of 7 m it is 3.6, and at a depth of 8 m it is 18.2. Further experimental studies with immersion of the supersensitive detector of hydrosphere pressure variations and the Valeport Mini SVP sound velocity profiler to depths of 10 m or more will make it possible to calculate the coefficient value more accurately.

## 5. Conclusions

To calculate the data conversion coefficient of the supersensitive detector of hydrosphere pressure variations, a number of experimental studies were carried out. The laser interference device, together with the Valeport Mini SVP sound velocity profiler, was installed in three bays of the Primorsky Territory at different depths. In Vityaz Bay, they were installed at a depth of 5 m; in Ulysses Bay in Vladivostok, the instruments were immersed to a depth of 7 m; and in Alekseev Bay, Popov Island, to a depth of 8 m. For all bays, different periods of sea waves, from several seconds to tens of minutes, were singled out. After estimating the amplitudes of pressure variations caused by sea pressure, the average values of the conversion factors for each of the depths were calculated. As a result of our calculations, we found that with an increase in depth, the value of the conversion coefficient also increases, but not according to the linear law. Thus, for a depth of 5 m, it is 11.6 Pa/V; for a depth of 7 m, it is 28.1 Pa/V; and for a depth of 8 m, it is 38 Pa/V. For more accurate plotting of the dependence graph, it is necessary to conduct such experiments at depths of 10 m and more. The obtained results allow calculating the amplitudes of sea waves in experimental studies using the supersensitive detector of hydrosphere pressure variations, which is capable of recording hydrosphere pressure variations in the frequency range from 0 (conditionally) to 1000 Hz with a measurement accuracy of 0.24 MPa at depths of up to 50 m.

For a more accurate plotting of the dependency graph, it is necessary to conduct such experiments at depths of 10 m or more. The supersensitive detector of hydrosphere pressure variations, with the Valeport Mini SVP sound velocity profiler attached to it, will be installed at depths of 10, 15, and 20 m in one or more bays. Further, fragments with oscillations from 4 s to 20 min will be singled out from the synchronous records of the instruments according to the scheme presented in this paper. For all these fragments of the records, the data conversion coefficients of the laser interference instrument will be calculated. Based on the available three points and the obtained ones, a curve of dependence of the conversion coefficient on the depth of immersion will be constructed, which will expand the range of tasks solved using the supersensitive detector of hydrosphere pressure variations.

## Figures and Tables

**Table 1:** Periods and amplitudes of wind waves, Vityaz Bay.

No	T, s	U, V	P, Pa	k, Pa/V
1	3.125	61.324	512.416	8.356
2	3.125	76.939	753.496	9.793
3	3.250	53.155	411.925	7.749
4	3.250	43.247	452.135	10.455
5	3.375	66.844	502.381	7.516
6	3.375	62.543	582.720	9.317
7	3.500	60.386	562.651	9.318
8	3.500	65.149	813.766	12.491
9	3.875	62.315	834.735	13.395
10	3.875	63.308	663.111	10.474

**Table 2:** Periods and amplitudes of swell waves, Vityaz Bay.

No	T, s	U, V	P, Pa	k, Pa/V
1	10.000	37.994	542.249	14.272
2	10.000	34.065	542.320	15.920
3	10.125	49.444	561.751	11.361
4	10.875	46.671	462.160	9.902
5	11.125	39.358	451.894	11.482
6	11.375	39.619	552.385	13.942
7	11.625	58.749	522.159	8.888
8	13.125	35.194	542.269	15.408
9	13.750	37.627	351.505	9.342
10	14.000	54.514	522.219	9.580

**Table 3:** Periods and amplitudes of infragravity waves, Vityaz Bay.

No	T, min	U, V	P, Pa	k, Pa/V
1	1.230	77.079	713.195	9.253
2	1.300	74.670	1034.635	13.856
3	1.340	72.071	904.050	12.544
4	1.480	15.144	217.454	14.359
5	2.180	71.799	876.416	12.207
6	5.400	96.976	865.045	8.920
7	5.450	92.958	1333.474	14.345
8	6.100	92.413	1367.526	14.798
9	6.080	98.052	1563.002	15.941
10	6.150	113.674	1028.508	9.048

**Table 4:** Periods and amplitudes of eigen oscillations of the bays, Vityaz Bay.

No	T, min	U, V	P, Pa	k, Pa/V
1	15.290	24.973	294.499	11.793
2	15.510	20.775	243.501	11.721
3	16.490	37.359	486.781	13.030
4	18.240	64.331	633.508	9.848
5	18.400	20.910	284.334	13.598
6	18.430	31.414	439.193	13.981
7	19.080	24.052	339.483	14.145
8	19.110	35.054	413.609	11.799
9	19.200	50.314	450.880	8.961
10	19.200	50.619	565.945	11.181

**Table 5:** Periods and amplitudes of wind waves, Ulysses Bay.

No	T, s	U, V	P, Pa	k, Pa/V
1	2.500	6.597	160.720	24.363
2	2.625	4.642	130.585	28.131
3	4.125	4.060	100.450	24.739
4	4.625	5.093	140.630	27.612
5	4.875	3.525	99.446	28.209
6	5.125	4.733	120.540	25.467
7	5.250	4.430	120.540	27.211
8	5.250	5.364	140.630	26.217
9	5.625	4.422	110.495	24.990
10	5.875	4.098	110.495	26.966

**Table 6:** Periods and amplitudes of swell waves, Ulysses Bay.

No	T, s	U, V	P, Pa	k, Pa/V
1	16.125	2.901	90.305	31.130
2	18.125	2.553	70.315	27.546
3	18.625	3.442	89.401	25.974
4	18.750	2.424	79.356	32.743
5	19.125	5.004	130.585	26.095
6	20.125	4.118	110.495	26.830
7	21.750	3.424	99.446	29.041
8	22.000	3.113	99.446	31.950
9	24.250	2.705	90.405	33.424
10	25.000	3.156	79.356	25.146

**Table 7:** Periods and amplitudes of infragravity waves, Ulysses Bay.

No	T, min	U, V	P, Pa	k, Pa/V
1	1.080	4.022	120.540	29.972
2	1.200	6.037	150.675	24.961
3	1.230	3.404	100.450	29.508
4	1.260	4.398	120.540	27.406
5	1.280	6.952	170.765	24.563
6	2.580	5.587	170.765	30.563
7	4.220	7.016	180.810	25.771
8	4.230	7.387	251.125	33.994
9	4.440	7.777	210.945	27.125
10	5.220	8.778	261.170	29.753

**Table 8:** Periods and amplitudes of eigen oscillations of the bays, Ulysses Bay.

No	T, min	U, V	P, Pa	k, Pa/V
1	9.840	1.450	48.484	33.437
2	10.000	2.557	76.028	29.733
3	10.490	4.868	122.850	25.236
4	11.290	3.635	95.158	26.178
5	12.010	1.972	57.837	29.328

**Table 9:** Periods and amplitudes of wind waves, Alekseev Bay.

No	T, s	U, V	P, Pa	k, Pa/V
1	2.750	0.995	40.134	40.336
2	3.125	1.116	50.208	45.009
3	4.000	0.897	40.180	44.793
4	4.125	1.271	40.180	31.605
5	4.750	1.297	50.125	38.655
6	5.125	1.587	50.225	31.650
7	5.500	0.882	40.174	45.549
8	6.375	1.502	50.225	33.438
9	6.500	1.294	60.270	46.576
10	6.875	1.288	40.180	31.196

**Table 10:** Periods and amplitudes of swell waves, Alekseev Bay.

No	T, s	U, V	P, Pa	k, Pa/V
1	10.875	0.958	31.045	32.391
2	11.000	1.347	60.350	44.817
3	11.000	1.207	60.291	49.959
4	11.750	1.296	40.269	31.074
5	11.750	1.800	70.285	39.041
6	12.000	1.235	40.259	32.598
7	12.625	1.087	50.322	46.303
8	12.875	1.985	80.385	40.502
9	13.375	1.891	60.306	31.891
10	13.625	1.221	60.209	49.307

**Table 11:** Periods and amplitudes of infragravity waves, Alekseev Bay.

No	T, min	U, V	P, Pa	k, Pa/V
1	1.050	1.855	70.748	38.145
2	1.190	2.154	70.315	32.651
3	1.220	2.734	99.918	36.553
4	1.300	2.667	110.495	41.437
5	1.260	2.761	109.892	39.799
6	3.570	6.224	189.047	30.373
7	4.090	5.037	188.876	37.496
8	4.200	3.954	152.794	38.640
9	4.300	3.309	138.812	41.945
10	4.410	5.631	183.251	32.546

**Table 12:** Periods and amplitudes of eigen oscillations of the bays, Alekseev Bay.

No	T, min	U, V	P, Pa	k, Pa/V
1	9.010	1.839	58.045	47.204
2	9.300	3.752	120.791	31.877
3	10.430	3.619	115.357	32.088
4	10.460	2.988	95.893	31.562
5	11.210	2.235	105.483	32.190

## Data Availability

Third Party Data. Restrictions apply to the availability of this data.
